# Field based research in the era of the pandemic in resource limited settings: challenges and lessons for the future

**DOI:** 10.3389/fpubh.2024.1309089

**Published:** 2024-02-29

**Authors:** Rubina Mulchandani, Tanica Lyngdoh, Sheetal Gandotra, H. S. Isser, Rajinder K. Dhamija, Ashish Kumar Kakkar

**Affiliations:** ^1^Indian Institute of Public Health-Delhi, Public Health Foundation of India, Gurgaon, India; ^2^Academy of Scientific and Innovative Research (AcSIR), Ghaziabad, India; ^3^Division of Reproductive, Child Health and Nutrition, Indian Council of Medical Research, Department of Health Research, Ministry of Health and Family Welfare, Government of India, New Delhi, India; ^4^Institute of Genomics and Integrative Biology, Council of Scientific and Industrial Research (CSIR), New Delhi, India; ^5^Department of Cardiology, Vardhman Mahavir Medical College and Safdarjung Hospital, New Delhi, India; ^6^Department of Neurology, Institute of Human Behaviour and Allied Sciences, University of Delhi, New Delhi, India; ^7^Post Graduate Institute of Medical Education and Research (PGIMER), Chandigarh, India

**Keywords:** COVID-19, field research, remote research, telephonic survey, digital health, LMICs

## Abstract

The coronavirus pandemic that began in December 2019, has had an unprecedented impact on the global economy, health systems and infrastructure, in addition to being responsible for significant mortality and morbidity worldwide. The “new normal” has brought along, unforeseen challenges for the scientific community, owing to obstructions in conducting field-based research in lieu of minimizing exposure through in-person contact. This has had greater ramifications for the LMICs, adding to the already existing concerns. As a response to COVID-19 related movement restrictions, public health researchers across countries had to switch to remote data collections methods. However, impediments like lack of awareness and skepticism among participants, dependence on paper-based prescriptions, dearth of digitized patient records, gaps in connectivity, reliance on smart phones, concerns with participant privacy at home and greater loss to follow-up act as hurdles to carrying out a research study virtually, especially in resource-limited settings. Promoting health literacy through science communication, ensuring digitization of health records in hospitals, and employing measures to encourage research participation among the general public are some steps to tackle barriers to remote research in the long term. COVID-19 may not be a health emergency anymore, but we are not immune to future pandemics. A more holistic approach to research by turning obstacles into opportunities will not just ensure a more comprehensive public health response in the coming time, but also bolster the existing infrastructure for a stronger healthcare system for countries.

## Introduction

In December 2019, the city of Wuhan in China reported the first human case of the novel coronavirus infection caused by the SARS-CoV2, a disease that we now know as COVID-19. The outbreak spread globally soon after, with the World Health Organization (WHO) declaring COVID-19 a global pandemic on March 11, 2020 (1). Three years on, the total number of confirmed COVID-19 cases (as of March 10, 2023) worldwide are more than 676 million, while the total number of confirmed deaths stand close to 6.9 million (2). The pandemic has resulted in huge economic losses, a breakdown of the fragile health infrastructure especially in the lower- and middle-income countries, and a significant increase in poverty and unemployment, above and beyond the high mortality and morbidity rates in the affected populations (3–7). The effects of COVID-19 have thus been far-reaching, and we continue to grapple with multiple concerns encountered in this “new normal”.

One of the greatest unrivaled challenges faced by the medical and scientific communities has been the disruption of non-coronavirus related clinical and public health research activities with trials and field studies getting delayed or prematurely concluded (8–11). Protocols like maintaining physical distancing, restricting travel and avoiding gatherings and meetings to lower the risk of transmission of this highly infectious viral disease, as well as redirection of existing funds for pandemic research, have made field-based research work involving human participants in healthcare settings like hospitals and clinics as well as in the community rather challenging (10, 11). Although non-COVID research took a hit across countries, low-middle-income countries (LMICs) have suffered a greater impact due to amplification of existing difficulties (12). As a response to this, researchers across the globe have shifted to virtual or remote methods of collecting study data during the pandemic (13, 14). This involves telephonic and tele-conference methods, as well as web-based applications to communicate with the participants, while ensuring the wellbeing of everyone involved in such studies. Digital approaches and the use of technology have thus gathered immense momentum in the last 2 years since they allow people to participate without worrying about exposing themselves to the infection. Such methods make it easier for the researchers to continue their work safely, while adhering to COVID-19 appropriate mandates. Various remote methods have been in use in high-income countries, such as interactive voice response (IVR), computer-assisted telephone interviews (CATI), short message service (SMS) and video conferencing (via zoom/skype), for both qualitative and quantitative purposes (13, 15). Due to lower levels of education and internet access and availability issues in spite of extensive use of mobile phones in low and middle income countries (LMICs), telephonic methods are more common and preferred over online internet-based methods (16). However, such approaches come with their own set of complications, especially in developing nations. Such challenges have been further augmented by COVID-19 (17).

Our paper aims to document the significant impediments to epidemiological research amidst the pandemic, in a resource-limited setting, based on our experience of conducting a hospital-based observational study in North India, substantiating them with existing evidence in this regard. We also propose ways to address some of these setbacks and suggest feasible solutions. Our research study is an ongoing prospective cohort involving in-person recruitment of patients on statins from the cardiology outpatient department of a tertiary care hospital in Delhi, and subsequent remote data collection telephonically, for a 2 year follow-up period with data obtained at baseline, and at the end of the first and second years.

## Barriers to conducting field-based research during the COVID-19 pandemic

### Economic constraints, lack of awareness and skepticism

The health system in the LMICs comprises of both private and public health care facilities and a significant proportion of the population opts for private health centers (18, 19). According to a few published reports, the private sector accounts for a considerable share of healthcare services in developing economies and caters to the lower income groups as well (40%, 57% and 62% in the African, South-East Asian, and Western Mediterranean regions) (20–22). The population groups that cannot afford private healthcare services are thus dependent on large public tertiary care centers offering medical care at a highly subsidized cost (18, 19, 23). Thus, in developing nations, a significant proportion of the patients at tertiary care centers belong to the lowest socio-economic strata.

The literacy levels in patients are also rather low, with studies reporting a 30%−45% prevalence of low to no education across LMICs (24–28). This in turn leads to a sub-optimal level of health literacy, i.e., their understanding and knowledge of their disease condition and medications. Limited awareness of clinical research and its relevance have also been reported as deterrents to research interest and participation in a few studies (29–34).

The pandemic has exacerbated this challenge. Remote interactions are now more feasible and safer as compared to in-person interactions, and it is difficult to explain things telephonically and convey the point across as effectively as one would, in a face-to-face setting (35). Gaps in communication act as hindrances to data collection. Also, building trust without an in-person interaction requires both patience and time, since a phone call doesn't offer the same personal touch (13, 36).

### Unavailability of digital records and the use of paper-based prescriptions

Large government/public hospitals have a high burden of patients, a heavy footfall in their Out Patient Departments (OPD) and limited resources (37, 38). The existing infrastructure makes it difficult to establish and maintain digital records or online databases for admitted patients and OPD patients in most such facilities. Unlike the west, the implementation and use of electronic medical record (EMR) systems in the LMICs remains rather minimal, and is limited to a handful of private tertiary care centers, while an overwhelming majority in the private and public sector still work with paper-based records (39–44). Additionally, the paper-based prescriptions are usually available only with the patient (45). Prescriptions and other documents like test reports and discharge summaries tend to get lost, torn or misplaced, and unavailability of records makes it impossible to track patients or obtain their history remotely (46).

### Ensuring correctness of contact information

Since remote modes of data collection are largely dependent on establishing contact through a mobile phone in resource limited settings, the contact information provided to the researcher is of prime importance. However, the numbers provided for the call may turn out to be erroneous or out of service, leaving the investigators with no choice but to drop the participant. At times the contact number may stop functioning due to inability of patients/caregivers to recharge/top up the talk time given the financial constraints exacerbated by the pandemic. This also gives rise to the need for multiple contact points within the participant's family, so that if the primary phone number turns out to be incorrect or non-functional, contact can still be established through alternate numbers. This is a time-intensive activity since the respondents' family/friends need to be contacted first, in order to be able to communicate with the participant (36).

### Dependence on smart phones and instant messengers

Remote data collection methods involve the use of a smartphone with an internet connection and instant messaging apps like WhatsApp. This can be used to obtain drug prescriptions, biochemical test reports, scans and other such source documents from the patient. According to a 2023 report, more than half of the world's current population, now owns a smartphone, with 4.3 billion users (47). WhatsApp messenger is also gaining momentum for use in population-based surveys and provides new opportunities for enhanced communication and engagement during fieldwork (48). However, its use is currently limited in LMICs and both its potential and concerns with respect to data collection in health research need further exploration (49). Also, roughly 3 billion people, about 38% of the world population, despite living in mobile broadband network areas, do not use the Internet (47). Therefore, participants with limited means, especially those in the older age groups, may not possess a smart phone/WhatsApp, or may not be well versed with its functioning and correct usage (50, 51). In such cases, gathering data becomes an arduous task (36, 52).

### Connectivity and network issues in rural areas

The economy has taken a massive hit as a result of the pandemic. This has led to a significant increase in unemployment, which in turn has pushed the working class into poverty. Consequently, a large number of people belonging to the lower socio-economic strata had to migrate back to their ancestral homes often in remote rural locations (53–55). This has inevitably affected data collection procedures adversely. Network and connectivity play a major role in carrying out remote research work. City outskirts, suburban and rural areas may not have adequate network coverage which results in weak signals, call drops and patchy internet connectivity (13, 16). Each interview with a participant residing in such a location takes longer and usually involves more than one call making it a time-intensive endeavor. These disturbances and interruptions also hamper the overall quality of the data collected during remote telephone-based interviews (36). Virtual modes of collecting research data have compounded the already existing digital divide, putting the economically weaker participants at a disadvantage in many aspects (14, 56).

### Decreased patient footfall in the hospitals

There is often a dearth of tertiary care health services in low-and middle income countries (LMICs). Tertiary care hospitals even when available, are present only in the major cities (57, 58). Hence, they cater to patients not just from the same city, but also from various neighboring cities and regions across the country. In the wake of COVID-19, health related travel went down, unless there was a medical emergency or a health condition that required immediate attention. This could be to avert the risk of infection and to avoid spending money on inter-city travel at a time when finances are rather limited. As a consequence, the total number of patients visiting these health facilities was much lesser than it used to be pre-pandemic (59–62).

Lesser footfall means a smaller sampling frame to choose from. This leads to longer periods of recruitment and contributes to delays in study conduct. Progress of studies requiring in person follow ups can be expected to be hampered similarly. Transport and distance related concerns have always been barriers to participating in field based research that have been further compounded during the pandemic times (32).

### Issues with privacy

Unlike in-person interviews, where the investigator can choose an appropriate setting for the patient to be in, interviews conducted remotely do not offer the same flexibility in terms of the surrounding environment of the participant (63). Often, participants when called, are at home, sitting with their family members. Space constraints and overcrowding with several people living together makes it almost impossible to talk to the participant privately. Sometimes participants find a place outside their homes where they may be joined by a neighbor or a friend (13, 14). In face-to-face settings, the researcher has significant control on the environment and can ensure privacy at all times. However, this onus is placed on the participants in remote research (13). Such situations act as obstacles to data collection which ideally requires a silent area. It thus becomes difficult to ensure confidentiality and accuracy of responses which affects validity of the data and continued participation (34).

### Ethical challenges

Conducting research during a pandemic is essential as well as necessary, but the appropriateness of the same may be debatable. Subjecting the participant to an extensive interview or survey during unprecedented times when people are struggling with a deadly virus, monetary losses and other peculiar disruptions in the wake of a global pandemic could pose a moral dilemma. Additionally, obtaining verbal consent in remote research work comes with its own set of challenges, in terms of maintaining a record of the consent obtained, while being mindful of privacy and mitigating the risk for coercion, in order to maintain the voluntary participation requirement. This becomes an even more important consideration when dealing with vulnerable groups or studies involving sensitive topics (17). Remote data collection may also put a greater responsibility on the participants, in terms of getting their phones recharged, figuring out the use of WhatsApp or other remote data collection apps, and finding an appropriate space at home to respond to the investigator's calls. On the contrary, such methods relieve the participants of the burden associated with spending time and the opportunity cost to travel to the study setting/hospital, and in many cases, missing out on their daily wages. Such issues often find themselves at the center of debate and discussion. The pros and cons of this conundrum need to be weighed for every research study, following which it should be dealt with in a manner that is sensitive, does no harm to participants while also ensuring that science and biomedical research are not unduly hampered by the pandemic (13).

### Increased frequency of non-response and higher attrition rates

Conducting research using remote methods like mobile phones can lead to a higher non-response rate in the study population (64, 65). Response rates ranging from 40 to 55% for telephonic surveys and interviews have been previously reported in literature (66–70). Higher non-response has been correlated to older, less affluent and less educated individuals, and is found to be affected by connectivity and low internet bandwidth issues too (65–68, 71). This becomes even more relevant when it comes to living in small crowded spaces, joint phone ownership in the family and limited availability of resources to maintain digital connectivity, along with mistrust in unknown numbers and misconstruing calls as being phishing/spam (13, 16). Also, the older adults are sometimes uncomfortable speaking over the phone and prefer a face-to-face discussion which could be a reason for their refusal to participate. At times, this translates to women and the aged being under-represented owing to lack of autonomy and independence in the household (13). In some patriarchal settings, the male spouse may choose to respond on behalf of the female which affects the accuracy of the answers (13, 72). Moreover, since mobile phones are the predominant mode of communication, tracking participants down for follow-up investigations and interviews is an uphill task too. Once they are aware of how the telephonic survey would go, some of them stop taking follow-up calls saying they don't have time for another round of interview, or that they don't understand the reason for a second call (13, 16). Also, a majority of mobile phone users subscribe to prepaid connections (73, 74), which are more likely to get discontinued, and thus could immediately cut the participant off from the researcher. Thus, remote surveys might run the risk of a higher dropout rates and greater loss to follow-up, as compared to in-person studies (75). It is possible that such studies have a slight overrepresentation of people belonging to the higher socio-economic groups, those with access to individual smartphones and ability to use the internet, those with higher literacy and those living in relatively less crowded homes (13, 76).

### Maintaining respondent engagement and interest

Ensuring the interest and attention of the respondents over a phone call is rather demanding. Additional efforts need to be made to keep participants engaged since this can affect the overall quality and accuracy of the collected data (64). This pertains to very young and very old individuals, who may get bored or lose interest and hang up in the middle of the interview, leaving responses incomplete. Longer interactions/questionnaires may further fatigue or distract the participants, leading to the information captured being unreliable and/ or invalid (16, 36).

## Possible solutions for conducting field based research in a pandemic scenario

Clinical research is vital to reducing disease burden, enhancing health, and increasing the overall quality of life in populations. It also provides insights into disease pathology and epidemiology that can help scientists and researchers tackle new diseases and improve patient outcomes (77, 78). Thus, research becomes even more essential during a public health crisis. Even as the COVID-19 pandemic subsides, the challenges of conducting research, especially remotely, continue to exist. We have provided insights based on our experience with quantitative research. However, the restrictions that come with a pandemic have equally affected qualitative research as well (79–81). In fact, there are several overlapping issues affecting both the approaches to health research conduct, while some remain unique to each methodology (82). The following section describes possible solutions to address some of the barriers highlighted above (Table 1).

**Table 1:** Selected barriers and possible solutions for conducting field research during a pandemic.

**Barriers**	**Possible solutions**
Economic constraints, lack of awareness and skepticism	Optimized fund allocation Improving health literacy Encouraging science communication Enhancing participation in scientific research
Unavailability of digital records and the use of paper-based prescriptions	Digitization of patient health records
Ensuring correctness of contact information	Digitization of patient health records
Decreased patient footfall in hospitals	Digitization of patient health records and establishing robust remote research systems
Issues with privacy and ethical challenges	Reminding participants of research context Ensuring flexibility in timing of communication Transparency regarding risks involved and process of data collection and utilization
Increased frequency of non-response and higher attrition rates	Improving health literacy Encouraging science communication Enhancing participation in scientific research Optimized fund allocation Utilizing technological advancements
Maintaining respondent engagement and interest	Improving health literacy Encouraging science communication Enhancing participation in scientific research Addressing research hesitancy and building trust

### Improving health literacy

The World Health Organization defines health literacy as ‘the achievement of a level of knowledge, personal skills, and confidence to take action to improve personal and community health by changing personal lifestyles, and living conditions' (83). In other words, it is the ability of a person to make sense of health related information so as to implement the same in their routine activities, and augment their quality of life (84). Health literacy rates in the developing countries are significantly low, owing to inadequate education, economic constraints, and other socio-cultural barriers (84, 85).

Improving health literacy among populations can enable people to play a more active role in their treatment and overall health. Targeted health interventions focused on populations with limited or no health literacy can improve their understanding of their health, reduce skepticism toward scientific research, enhance treatment compliance and strengthen doctor-patient relationships (86). This would ultimately augment interest in research participation and support remote research activities in the long term. Better knowledge of their own health, clinical care and the relevance and need for research could improve response rates and encourage willingness of the participants to contribute both in-person and virtually.

This would require improvements in the existing health infrastructure to support access to relevant health information, more manpower for engagement with medical professionals as well as the general public, and encouraging the practice of science communication in healthcare for communicating information to the patients in a language and manner they understand. Easy to read infographics, posters and pamphlets prepared in local languages, training health educators for interacting with communities, integrating health literacy in the educational curriculum in schools, and sensitizing researchers, scientists and medical professionals about this issue have been shown to be effective in this regard (84, 87). However, it is important to note that health literacy is a complex issue which is a function of various systemic factors like linguistic, social and cultural barriers, poverty and lower standards of living, gender disparities, as well as shortcomings of the current education system, which in turn result in lack of basic education and sub-optimal literacy levels overall (88–92). Addressing these fundamental concerns through policy level changes and national reforms, with various stakeholders working synergistically, is a starting point that would eventually contribute to improved health literacy levels as well.

### Encouraging science communication

Science communication (SciComm) has gathered a lot of momentum over the years, as a result of growing interest of educators, scientists and communication experts in this field. It is based on the broad concept that distinguishes information availability and accessibility. Readily available medical information in research papers, textbooks, newspapers, may not be accessible to the layperson. Also, accessibility itself does not ensure usability. Technical jargon, unfamiliar vocabulary and complex texts can act as major hindrances to uptake of information by the general population. Science communication aims to bridge this knowledge gap, by making important information available and accessible to the public, through simpler narratives translated in multiple languages, for easier consumption (93–96). A good example of SciComm is clinicians communicating information about a disease condition to a patient (explanation of their illness, the treatment regimen, adverse-effects if any, and precautions to be taken) in a simplified manner and in the vernacular language specific to that region. It is an ecosystem that encompasses numerous stakeholders, each with a designated role, and involves multiple communication pathways -digital, verbal, visual amongst others (93, 97).

Changes at the individual as well as the policy level can contribute to improved health literacy through SciComm. There is a dearth of literature on the effect of various interventions on health literacy rates in resource limited settings, warranting the need for extensive research to understand economic implications of low literacy rates and the cost-effectiveness of various interventions- traditional/learning based (booklets, pamphlets), art based (storytelling), interaction based (peer-support programs) and technology based (digital devices and websites) (88, 98, 99). However, evidence from the developed world settings does suggest that higher literacy could prove to be cost-effective since lower health literacy levels are associated with higher medical costs (100, 101).

### Digitization of patient health records

An electronic health record (EHR) is a collection of medical records of a person that are created during a clinical event and get accumulated over their lifetime. Maintaining an electronic database helps keep a record of important medical information and history of the patient, avoid repeat investigations and improve the overall therapeutic experience for both care providers and receivers. The public sector IT system needs improvements in terms of internet speed and connectivity issues. Apart from data protection concerns, setting up infrastructure for digital systems is resource intensive and requires personnel (102). Other challenges that hamper the broader implementation of digital record systems in healthcare include limited financial backing, lack of processes for data integration, inadequate training and capacity building, low education levels, legislation and policy gaps, and concerns with cyber security laws, ethics and regulatory bottlenecks (103–106). Filling these lacunae is a herculean task and would require concerted efforts over time but can have a substantial positive impact on public health research and contribute to more robust evidence synthesis. Digital records become even more essential in remote research where participants might be required to furnish information through mobile phones. Availability of medical documents and other records in an online format could enable easy access for participants as well as easy sharing with the study investigators, ensuring completeness of the medical data obtained from each participant.

As part of the above, shifting to e-prescriptions can make doctor-patient consultations a much more seamless experience. Paper based prescriptions are prone to errors, can have handwriting issues, and run the risk of getting lost or misplaced, leading to permanent loss of crucial patient information and disease history (107–109). Some physicians have also suggested incorporating printed terms for “Morning,” “Afternoon,” and “Evening” in different local languages on the prescription sheet to overcome the language barrier (110). Keeping a scanned copy of the patient prescription with the consulting doctor/hospital is also believed to be a useful way of ensuring that a record of the patient history exists with the hospital in case the patient loses or forgets to carry it with them (110). Additionally, there is literature evidence to show that hospitals with electronic patient health records incur lower costs due to fewer errors and a more streamlined management system (111).

However, one needs to remember that the transition from paper based records to electronic records can only happen in phases, with establishments gradually shifting to a hybrid mode before operating in a paperless fashion. Even then, the paper based approach has its own advantages that cannot be overlooked or rendered redundant and while digital technology is the future, offline documentation can always serve as a backup repository for data storage.

### Enhancing participation in scientific research

People's willingness to participate in a study, whether hospital-based, field-based or remote, is one of the most pivotal aspects of clinical research. Acknowledging systemic and individual level hurdles in this regard is the first step toward enhanced participation rates at the start of the study and reduced attrition while it is ongoing. Systematic reviews conducted in the past have suggested a few factors that could effectively favor participation in research (34, 95, 96, 112).

#### Providing clarity regarding short-term or long term benefits for the participant

Patients are found to be more likely to participate in research studies if they are convinced that the output will benefit their health. It is essential to be transparent as well as realistic with participants in terms of what they can expect from their involvement (34, 95, 96, 112). Additionally, efforts should be made to make them understand that research is not the same as medical care and immediate treatment benefit may not be a possibility (95).

#### Instilling a sense of altruism

The feeling of being able to contribute to collective good has been found to influence patients' decision to participate in research in some cases. Making them aware of the larger goal of improved therapeutic experience and enhanced clinical care for future patients could serve as an impetus for participation (113, 114).

#### Sharing details of any risks involved

Adequately informing participants of any major or minor risks involved can ensure greater trust in the research process, which in turn could positively impact participation rates. This requires detailed patient information sheets and availability of the study team/personnel for answering questions and addressing apprehensions (34, 96).

#### Maintaining transparency in data collection and utilization processes

Various approaches to garner greater confidence in the research and its findings have been suggested in literature, including sharing of study data where applicable, dissemination of results among the participants, and keeping them updated about the study progress along with other stakeholders (34, 95, 96, 112). The language and terminology used for communicating such information plays a crucial role in communication (34, 95, 96, 112).

#### Facilitating access to the healthcare provider

In tertiary care public health facilities, the doctor-patient ratio is highly skewed, resulting in heavy patient loads in most outpatient as well as inpatient departments. Making efforts to provide participants better access to therapeutic care, especially where the clinician researcher is leading the study, could also serve as a significant impetus to continued participation and retention in the study (34, 95, 96, 112).

#### Addressing research hesitancy

Tackling skepticism about research among the patients is paramount. Alleviating their concerns with empathy and establishing the importance of their participation through regular engagement and communication can help avoid feelings of distrust and apprehension toward research (34, 95, 96, 112).

#### Building patients' trust in the researchers

Rapport building is an essential component of any clinical research study. Being available for the patients, providing them with a contact number they could use and setting aside some time to address any queries they may have, related to the ongoing research or the clinical care, could be a source of validation for them, lowering their reluctance to engage with the investigators over the course of the study (34, 95, 96, 112).

Further, remote data collection brings along some peculiar issues pertaining to privacy, the participant's overall understanding of the setting (since they are usually in their homes), establishment of initial contact, and internet and connectivity hassles. The following ways could ensure greater willingness to participate in such studies and lower the risk of attrition (14, 115).

#### Reminding them of research context

Remote research involves participants attending interviews from their homes, instead of being present in a formal setting. This can lead to them forgetting the purpose or context of the investigator's call. In such cases, reminding them of the purpose of the study can improve data quality while allowing participants to speak comfortably (14, 115).

#### Offering alternative times for communication

Participation in research while being at home means less stringent schedules for investigators to operate within. Additionally, pandemic related disruptions can further interrupt people's daily routines and data collection may not always happen as planned. Sending a reminder beforehand, as well as providing a different time slot based on the participant's convenience saves the researchers' time and the flexibility keeps the participant interested (14, 115).

#### Working closely with their friends/family

Establishing more than one level of contact could help minimize drop outs and loss to follow ups. Efforts to obtain contact information of a family member, or friend who is either a caregiver, accompanies the patient for their hospital visits or is involved in any other aspect of their treatment becomes important if access to the patient is getting difficult (14, 115). Communication with a family member could also ensure greater trust from their end.

#### Optimizing fund allocation

Since remote research involves much lesser travel to the hospital/clinical setting, provisions could be made to divert that component of the study grant toward providing call and internet services to the participants (14, 115). Enhanced access to technology through resource optimization can streamline the process of study data collection considerably and ensure continued participant engagement (14, 115).

#### Utilizing technological advancements

A remote research setting may not always allow immediate communication with the participant. Technological features like voice notes in WhatsApp can come in very handy in situations when a voice call is not feasible. Besides, text reminders for upcoming or missed follow-up calls can be helpful in ensuring participant availability at scheduled times (14, 115). This could foster continuity in research and can help keep the participants engaged during follow-ups.

## Conclusion

The pandemic has significantly altered the world we live in, bringing in a multitude of changes in various aspects of our routine lives. This has inevitably affected the way we conduct field-based research activities as well. Some of the challenges are unfamiliar, while others have just resurfaced or been magnified. However, the myriad of issues associated with carrying out primary research also bring opportunities to work differently and perhaps improve and strengthen the existing systems in place.

Remote research comes with its own set of concerns, but can also be highly effective in organizing routine surveillance measures for timely capture of health-related data. Addressing the barriers highlighted above through leveraging technology, investing in health infrastructure, and facilitating greater awareness can modify our overall approach to research.

The worst of the COVID-19 pandemic has come to an end, but we are not immune to threats of future epidemics (116–120). Further, the lessons learned during this period can elevate existing research processes as a whole, fostering greater opportunities for scientific advancements in the coming time. Even when traditional methods of face-to-face research are possible, remote methods can help save time and money that could be employed elsewhere to improve the efficiency of field-based research. This transition may not be straightforward and would require being more receptive to incorporating newer ideas into our usual ways of conducting health research. However, the outcomes would be rather rewarding and worth the effort in the long run.

## Author contributions

RM: Conceptualization, Investigation, Methodology, Resources, Writing—original draft. TL: Methodology, Project administration, Supervision, Writing—review & editing. SG: Methodology, Project administration, Supervision, Writing—review & editing. HI: Methodology, Supervision, Writing—review & editing. RD: Methodology, Supervision, Writing—review & editing. AK: Conceptualization, Methodology, Resources, Supervision, Writing—review & editing.
